# Development and external validation of a prediction model for the transition from mild to moderate or severe form of COVID-19

**DOI:** 10.1007/s00330-023-09759-x

**Published:** 2023-07-05

**Authors:** Maéva Zysman, Julien Asselineau, Olivier Saut, Eric Frison, Mathilde Oranger, Arnaud Maurac, Jeremy Charriot, Rkia Achkir, Sophie Regueme, Emilie Klein, Sébastien Bommart, Arnaud Bourdin, Gael Dournes, Julien Casteigt, Alain Blum, Gilbert Ferretti, Bruno Degano, Rodolphe Thiébaut, Francois Chabot, Patrick Berger, Francois Laurent, Ilyes Benlala

**Affiliations:** 1 CHU Bordeaux, 33600 Pessac, France; 2 Univ. Bordeaux, Centre de Recherche Cardio-Thoracique de Bordeaux, 33600 Bordeaux, France; 3 Centre de Recherche Cardio-Thoracique de Bordeaux (U1045), Centre d’Investigation Clinique, INSERM, Bordeaux Population Health (U1219), (CIC-P 1401), 33600 Pessac, France; 4 “Institut de Mathématiques de Bordeaux” (IMB), UMR5251, CNRS, University of Bordeaux, 351 Cours Libération, 33400 Talence, France; 5 MONC Team & SISTM Team, INRIA Bordeaux Sud-Ouest, 200 Av Vieille Tour, 33400 Talence, France; 6 Pôle Des Spécialités Médicales/Département de Pneumologie, Université de Lorraine, Centre Hospitalier Régional Universitaire (CHRU) Nancy, Service de Radiologie Et d’Imagerie, Nancy, France; 7 Faculté de Médecine de Nancy, Université de Lorraine, Institut National de La Santé Et de La Recherche Médicale (INSERM) Unité Médicale de Recherche (UMR), S 1116, Vandœuvre-Lès-Nancy, France; 8 Department of Respiratory Diseases, Arnaud de Villeneuve Hospital, Montpellier University Hospital, CEDEX 5, 34295 Montpellier, France; 9 PhyMedExp, University of Montpellier, INSERM U1046, CEDEX 5, 34295 Montpellier, France; 10 Pneumology Clinic, St Médard en Jalles, France; 11 France Service de Radiologie Diagnostique Et Interventionnelle, Université Grenoble Alpes, CHU Grenoble-Alpes, Grenoble, France

**Keywords:** COVID-19, Tomography, X-ray computed, Clinical decision rules, Artificial intelligence

## Abstract

**Objectives:**

COVID-19 pandemic seems to be under control. However, despite the vaccines, 5 to 10% of the patients with mild disease develop moderate to critical forms with potential lethal evolution. In addition to assess lung infection spread, chest CT helps to detect complications. Developing a prediction model to identify at-risk patients of worsening from mild COVID-19 combining simple clinical and biological parameters with qualitative or quantitative data using CT would be relevant to organizing optimal patient management.

**Methods:**

Four French hospitals were used for model training and internal validation. External validation was conducted in two independent hospitals. We used easy-to-obtain clinical (age, gender, smoking, symptoms’ onset, cardiovascular comorbidities, diabetes, chronic respiratory diseases, immunosuppression) and biological parameters (lymphocytes, CRP) with qualitative or quantitative data (including radiomics) from the initial CT in mild COVID-19 patients.

**Results:**

Qualitative CT scan with clinical and biological parameters can predict which patients with an initial mild presentation would develop a moderate to critical form of COVID-19, with a c-index of 0.70 (95% CI 0.63; 0.77). CT scan quantification improved the performance of the prediction up to 0.73 (95% CI 0.67; 0.79) and radiomics up to 0.77 (95% CI 0.71; 0.83). Results were similar in both validation cohorts, considering CT scans with or without injection.

**Conclusion:**

Adding CT scan quantification or radiomics to simple clinical and biological parameters can better predict which patients with an initial mild COVID-19 would worsen than qualitative analyses alone. This tool could help to the fair use of healthcare resources and to screen patients for potential new drugs to prevent a pejorative evolution of COVID-19.

**Clinical Trial Registration:**

NCT04481620.

**Clinical relevance statement:**

CT scan quantification or radiomics analysis is superior to qualitative analysis, when used with simple clinical and biological parameters, to determine which patients with an initial mild presentation of COVID-19 would worsen to a moderate to critical form.

**Key Points:**

*• Qualitative CT scan analyses with simple clinical and biological parameters can predict which patients with an initial mild COVID-19 and respiratory symptoms would worsen with a c-index of 0.70.*

*• Adding CT scan quantification improves the performance of the clinical prediction model to an AUC of 0.73.*

*• Radiomics analyses slightly improve the performance of the model to a c-index of 0.77.*

**Supplementary information:**

The online version contains supplementary material available at 10.1007/s00330-023-09759-x.

## Introduction

Few patients infected with coronavirus disease 2019 (COVID-19) rapidly develop acute respiratory distress leading to respiratory failure, with high short-term mortality rates [1]. However, only 5% of patients infected with COVID-19 experienced this pejorative evolution [2]. Despite the vaccines, the pandemic is not over yet and a progression from a mild to moderate or severe form could not be excluded for at-risk subjects [3]. However, there is still no reliable risk stratification tool for non-severe COVID-19 patients at admission especially among those with respiratory symptoms further overwhelming the health system [4]. Patients with a mild disease typically recover at home [5], especially, because there is no fully proven therapy for these mild COVID-19 to prevent adverse evolution [6]. Nevertheless, new expansive strategies are emerging to prevent worsening from mild to severe COVID-19 [7], without distinction of a specific population likely to worsen.

Chest computed tomography (CT) is widely used to manage COVID-19 pneumonia because of its availability and rapid acquisition; it remains crucial in case of prolonged symptoms or new emergency signs. In addition to its role in early diagnoses during the first months of the pandemic, CT has a pivotal role in detecting complications such as thromboembolism [8], which can occur even in mild diseases [9]. Also, a prognostic role of chest CT has been reported in evaluating the extent of COVID-19 lung abnormalities [10, 11] while previous data have shown that it could predict severe outcomes [12–14]*.* Besides, clinical and biological parameters with artificial intelligence (AI) analyses of imaging data seemed to identify patients with severe outcomes in COVID-19 pneumonia [15]. However, most publications are based on small cohorts or severe forms [16–18], and there is no data about mild COVID-19, which are dramatically more frequent.

The goals of this multicenter study were to develop and validate clinical prediction models for the risk of progression from mild to moderate, severe, or critical COVID-19 combining simple clinical and biological parameters with qualitative or quantitative data (including radiomics) from the initial chest CT in mild COVID-19 patients with respiratory symptoms. This strategy could help to identify patients with low-risk worsening of SARS-CoV-2 pneumonia despite respiratory symptoms. Early identifying at-risk patients may address a major issue of a fair use of healthcare resources and would allow better screening for new expansive therapeutics to prevent a pejorative evolution of COVID-19.

## Materials and methods

### Ethics considerations

The study was conducted by international guidance and approved by a national Ethics Committee on 06/18/2020 (NCT04481620). The study conducts adhere to the TRIPOD statement recommended for developing and validating a prediction model. Study data were collected and managed using REDCap electronic data capture tools hosted at the University Hospital of Bordeaux [19].

### Study design and participants

In the development cohort (from 3 university hospitals in Bordeaux, Grenoble, and Montpellier and a private hospital in Bordeaux, France), patients were eligible if they were at least 18 years old, and had a first chest CT performed without injection of contrast agent for respiratory symptoms which led to highly suspicious or compatible according to standardized visual analysis of COVID-19. Besides, they should have either a biological diagnosis (RT-PCR) or a clinical suspicion (cough and/or dyspnea and/or fever and/or need to use oxygenotherapy as part of routine care) of COVID-19 at the time of the CT scan, between March 1, 2020, and May 5, 2021 (Figure S1). Non-inclusion criteria were patients with moderate or severe forms (defined as oxygenotherapy ≥ 3 L/min to obtain a SpO_2_ > 97%) or critical forms of COVID-19 (defined by the need for non-invasive or invasive ventilation and/or orotracheal intubation) on the date of the first chest CT. In the validation cohort (university hospitals in Nancy and Poitiers, France), eligibility criteria were similar, except that half of the patients had chest CT with a contrast agent injection.

### Outcome of interest and predictors

The composite outcome of significant clinical deterioration from a mild form of COVID-19 within 30 days after chest CT was defined by the occurrence of a moderate, severe (defined as oxygenotherapy > 5 L/min to obtain SpO_2_ > 97%), or critical form of COVID-19 or death [20]. The clinical and biological candidate predictors were selected from a literature review [8, 11, 21–23] and retrieved from the electronic medical records: age, gender, smoking, time elapsed since symptoms’ onset, and any pre-existing cardiovascular comorbidities such as coronary artery disease, hypertension, diabetes, obesity, respiratory diseases (COPD or interstitial lung disease), or immunosuppression. Clinical and biological parameters were collected in a 24-h window after CT scans.

### Validation cohort

We internally validated the model and estimated its performance in an independent validation cohort. Half of the validation cohort (*n* = 228) used participants with non-injected CT scans included between March 19, 2020, and January 28, 2021. The other half (*n* = 246) included participants with injected CT scans, between March 23, 2020, and April 23, 2021 (Figure S1).

### Chest CT

CT were acquired on 9 CT models (Table S1, supplemental data). The standardized report proposed by the French Society of Radiology (https://ebulletin.radiologie.fr/comptes-rendus-covid-19) was largely used by French radiologists across the participating centers. It includes a 5-scale score of severity (0% = absent; < 10% = mild; 10–25% = moderate; 25–50% = extended; 50–75% = severe; > 75% = critical) and a 4-point scale to categorize the risk of COVID-19: highly suspicious, compatible, not suspicious, and normal. Different patterns of COVID-19 lung lesions and their distributions were reported (ground-glass opacities, consolidations, and crazy paving) [24, 25].

### Quantitative assessment of CT

An AI-based software tool for chest CT analysis (*syngo*.via CT Pneumonia Analysis prototype) from Siemens Healthineers (Version 1.0.4.2) was used to assess the severity of COVID-19. It automatically segments the lungs/lobes and delineates lung opacities (ground-glass and consolidations) based on a convolutional neural network trained with data manually labeled by expert radiologists [26]. If needed, lung segmentation was adjusted manually. Low attenuation areas were defined when below -950 HU (LAA-950).

### Radiomics analyses of CT

Before extracting radiomics features, images were resampled on a 1 mm × 1 mm × 1 mm grid by PyRadiomics [27]. Preprocessing, harmonization, and normalization of features were scaled using the RobustScaler from scikit-learn framework [31], which removes the median and scales the data according to the quantile range.

Then, from the CT of each patient, PyRadiomics was used to extract two sets of radiomics features on two different ROIs for each patient: the COVID-19 lesion and the lung region not including the COVID-19 part. For each of these ROIs, we extracted a total of 107 radiomics features—with a bin width of 34—corresponding to first-order (*n* = 18), shape (*n* = 14), and second-order (gray-level co-occurrence matrix with 1-voxel distance to neighbors, gray-level run length matrix, neighborhood gray-level different matrix, and gray-levels zone length matrix, *n* = 75) groups of radiomics features. With the development cohort, the best model (i.e., a chain of preprocessing, selection, oversampling, model methods) was selected using mean values of the c-indexes metric over the repeated (*n* = 30) tenfold cross-validation [28]. The complete procedure was then retrained on the whole cohort and used for obtaining the predictions on the validation cohort. An additional filter was applied to the images before extraction (Laplacian of Gaussian filter with sigma = 2 mm), giving 186 extra features from each ROI. As these additional features did not significantly improve the results, we chose to discard them from our analysis. Thus, for each CT, 214 radiomics features were extracted.

An ablation study was also performed to investigate the importance of the different groups of imaging features. Results are shown in the two cohorts of the validation set (Table S5). “Lesion radiomics” considers only the set of radiomics features (107 features) extracted from the lesion; “Lesion + Lung radiomics” considers radiomics extracted from the lesion and parenchyma (214 features) and “Complete radiomics” was the model obtained with the full set of imaging features and clinical and biological features (226 features).

### Statistical analyses

A sample size of the development cohort was calculated using Riley and colleagues’ approach [29]. We hypothesized an incidence of significant clinical deterioration within 30 days at 20%; among mild COVID-19 [1, 2], 16 parameters included in the clinical prediction models and an expected Harrell’s c-index of 0.78 (Nagelkerke’s *R*^2^ of 0.25). The resulting sample size was at least 826 patients. For the external validation, we aimed to recruit at least one hundred clinical deterioration events for each validation cohort, as recommended by Vergouwe [30].

Three clinical prediction models were developed, combining clinical and biological factors with imaging parameters of increasing complexity: 5-scale score of severity based on CT visual assessment (model 1 or qualitative model); quantitative assessment of ground-glass, consolidation, and low attenuation areas on CT (model 2 or quantitative model); radiomics features (model 3) where 6 features were selected among the COVID lesions and lungs ROIs as the best features from the univariate analysis of the development cohort in each of the classical radiomics classes (shape-based features, first-order intensity features, and second-order intensity features), 2 features per group. In addition, we predefined the percentage of consolidation as a characteristic of interest to retain in the model.

The development of the prediction models was based on a logistic regression model whose response variable was defined by the outcome of interest described above. The missing data on outcome and predictors (Table S2) were handled as appropriate (supplemental data).

The predictive performances of the clinical prediction models were evaluated on samples of participants recruited in independent study centers (external validation). Finally, to estimate sensitivities, specificities, and predictive values of clinical prediction models, we dichotomized the outcome probability by using the median of the thresholds calculated in each imputed dataset in the development process to obtain a minimal desired specificity of 0.90 to select patients to avoid unnecessary hospitalizations/treatments.

### Development and exploration of machine learning model

We evaluated the predictive capacity of a larger set of radiomics features with machine learning algorithms. They were trained on the development cohort using repeated cross-validations. Model selection was performed on the development cohort and its performance was evaluated in the validation cohort. The computations were run in Python using the Scikit-learn platform [31]. Feature selection: first, the pairwise correlation between features was computed using Spearman rank correlation. When two features were highly correlated (correlation coefficient > 0.95), the last one was dropped (columns were randomly shuffled beforehand, and no significant change in performance was observed). Then, we kept the 50 best features from the univariate analysis (the procedure was done separately for each cross-validation fold, yielding potentially a different set of selected features for each fold).

## Results

### Baseline characteristics and outcomes of the development cohort

**Fig. 1:**
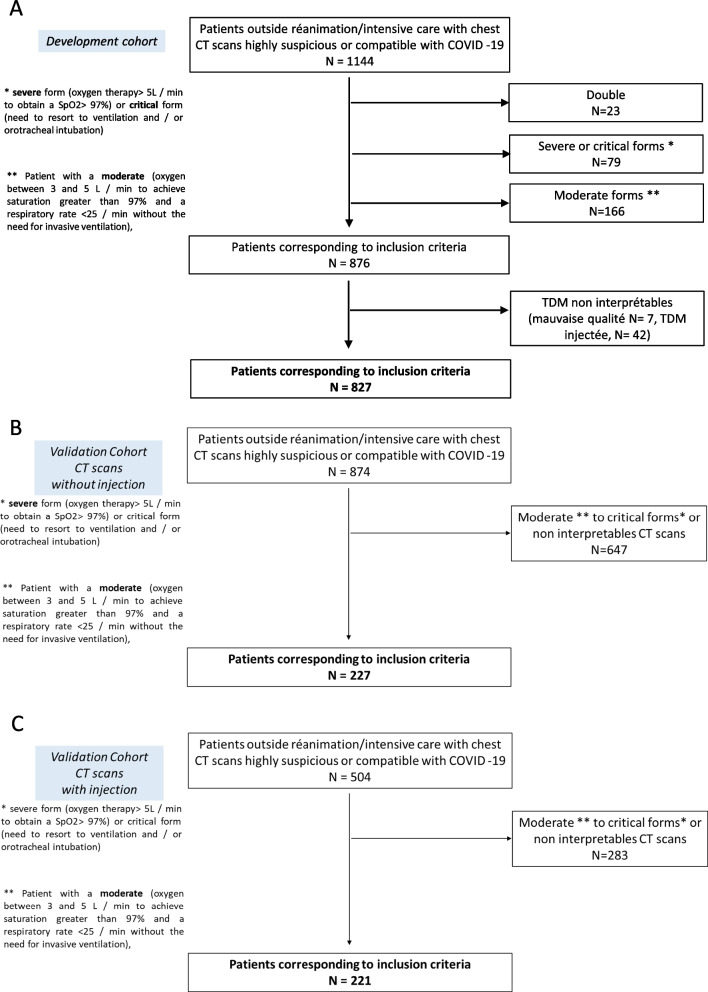
Flow chart of PREDICT-COVID in the validation cohort (**A**) and the development cohort with non-injected CT scans (**B**) and injected CT scans (**C**). CT, computed tomography

A total of 827 participants were included in the development cohort (Fig. 1). The study demographics are presented in Table 1 and Table S3. Briefly, mean age was 65.5 [IQR 54; 79] years; there were 495 (59.9%) men, with a median BMI of 27.4 [23; 30] kg/m^2^ and a median time between first symptoms and CT of 6 [2; 10] days. Comorbidities were mainly hypertension (373, 45.1%), obesity (178, 21.7%), and diabetes mellitus (170, 20.6%). Asthma and COPD affected respectively 9.3 and 8.5% of the population. A positive RT-PCR during the acute phase was reported for 461 (64.8%) participants. Mean lymphocyte level was 1.16 ± 1.35 G/L, CRP 86 ± 82 mg/L. CT features were distributed as follows: ground-glass opacities affecting 805 (97.3%). The extent of the COVID-19 suspected lesions were mild (182, 22.0%), moderate (389, 47.0%), extended (200, 24.2%), severe (52, 6.3%), or critical (4, 0.5%). Finally, 440 (53.2%) participants were graded highly suspicious for COVID-19 diagnosis, the others being compatible.

**Table 1:** Patient characteristics in the development and validation cohort

	Development cohort (*n* = 827)	Validation cohort (*n* = 474)	
		CT without injection (*n* = 228)	CT with injection (*n* = 246)
Clinical parameters			
Age (years), mean (SD)	65.5 (17.7)	66.6 (18.1)	64.6 (17.1)
Male gender, *N* (%)	495 (59.9)	110 (48.2)	126 (51.2)
BMI (kg/m^2^)	27.4 (6.3)	28.5 (6.6)	29.2 (5.5)
Time to symptoms onset (days), mean (SD)	7.4 (8.2) *	7.0 (5.6)	7.2 (4.3)
Active smokers, *N* (%)	77 (14.5)	24 (16.3)	7 (3.9)
Hypertension, *N* (%)	373 (45.1)	126 (55.3)	115 (46.7)
Coronary artery disease, *N* (%)	165 (20.0)	36 (15.8)	23 (9.3)
Obesity, *N* (%)	178 (21.7)	72 (36.0)	74 (39.2)
Respiratory diseases Asthma, *N* (%) COPD, *N* (%) Interstitial lung disease, *N* (%)	77 (9.3) 70 (8.5) 17 (2.1)	19 (8.3) 21 (9.2) 3 (1.3)	13 (5.3) 10 (4.1) 1 (0.4)
Diabetes, *N* (%)	170 (20.6)	59 (25.9)	62 (25.2)
Immunosuppression, *N* (%)	83 (10.0)	25 (11.0)	10 (4.1)
Biological parameters			
Lymphocyte level (g/L)	1.16 (1.35)	1.13 (0.93)	1.09 (0.58)
CRP (mg/L)	86.5 (82.1)	88.0 (75.8)	88.6 (73.4)
RT-PCR positive for COVID-19, *N* (%)	461 (64.8)	172 (80.4)	226 (95.8)
Radiological parameters			
Disease extent on CT scan Mild < 10%, *N* (%) Moderate 10–25%, *N* (%) Extended 25–50%, *N* (%) Severe 50–75%, *N* (%) Critical > 75%, *N* (%)	182 (22.0) 389 (47.0) 200 (24.2) 52 (6.3) 4 (0.5)	50 (21.9) 95 (41.7) 63 (27.6) 17 (7.5) 3 (1.3)	32 (13.0) 111 (45.1) 74 (30.1) 28 (11.4) 1 (0.4)
Outcomes			
Primary outcome in the 30 days	241 (32.3)	96 (43.2)	106 (43.3)
Moderate form Severe form Critical form Death	212 (28.4) 105 (14.1) 46 (6.2) 67 (9.3)	90 (40.5) 60 (27.0) 32 (14.5) 36 (16.3)	102 (41.8) 62 (25.4) 31 (12.7) 18 (7.4)

*COPD* chronic obstructive pulmonary disease, *CRP* C-reactive protein, *CT* computed tomography, *RT-PCR* real-time polymerase chain reaction, *SD* standard deviation

Missing data in Table S2

Significant clinical degradation was observed in 212 (28.4%) participants (Table S2). Severe and critical forms occurred respectively in 105 (14.1%) and 46 (6.2%) participants. The 30-day mortality rate was 9.3%, with a mean time from COVID-19 diagnosis of 11.5 (± 8.8) days (Table 1).

### Baseline characteristics and outcomes of the external validation cohort

A total of 474 patients were included from two independent centers (in the external validation cohort (228 patients with non-injected and 246 with injected CT, Fig. 1). Clinical characteristics were similar, as shown in Table 1, except for the gender with fewer men, a higher rate of obesity in both validation cohorts and more occasional smokers in the validation cohort with injected scans. A significant clinical degradation occurred in 90 (40.5%) participants from the non-injected validation cohort and 102 (41.8%) participants from the injected validation cohort. The 30-day mortality rate was 16.3% in the non-injected validation cohort and 7.4% in the injected validation cohort (Table 1).

### Performance of the qualitative model (model 1)

**Fig. 2:**
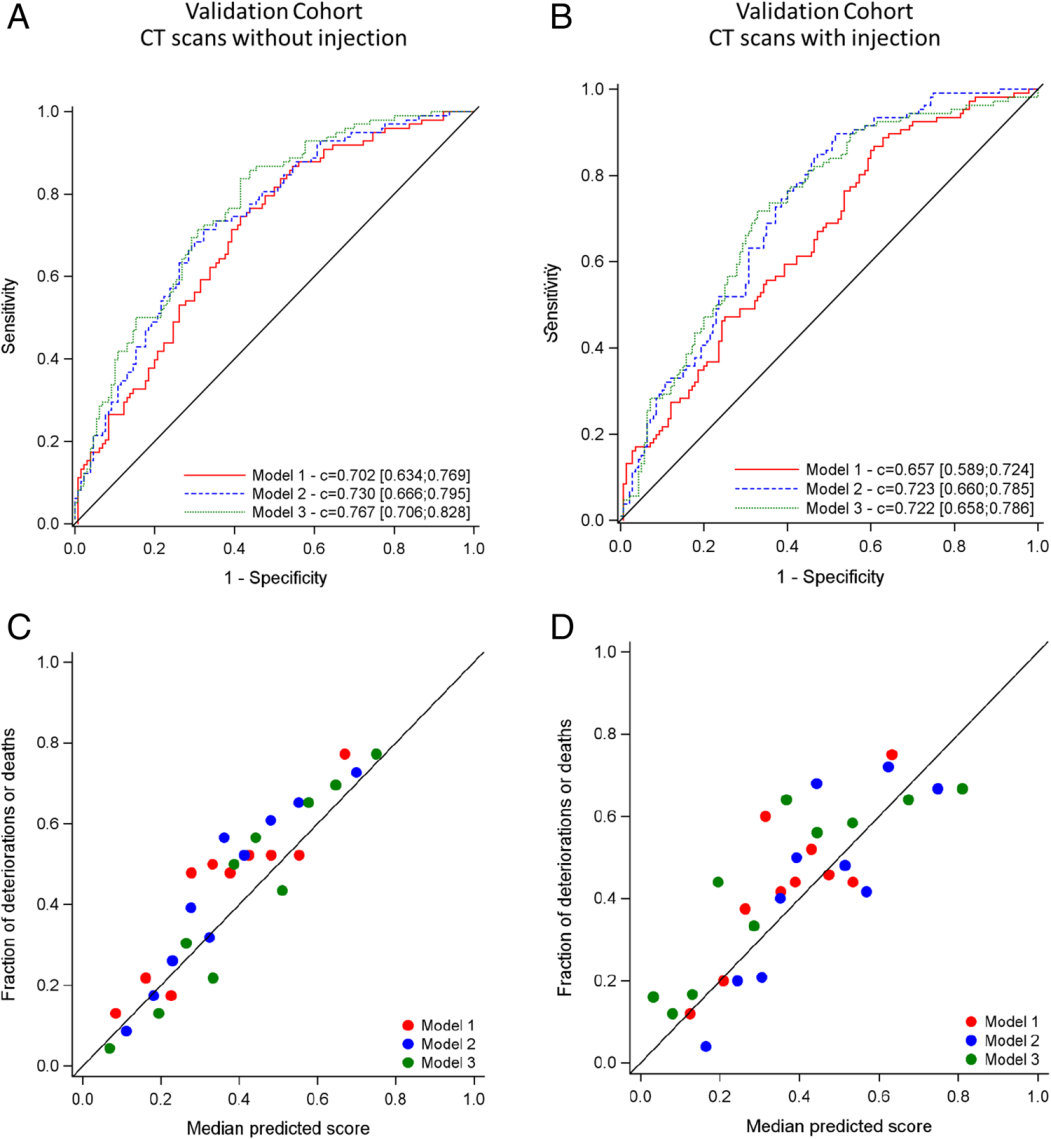
Performance of the qualitative (model 1), quantitative (model 2), and radiomics model (model 3), assessed by c-index representation (**A**) for non-injected CT scans and (**B**) for injected CT scans. The calibration of prediction models was also studied for non-injected CT scans (**C**) and injected CT scans (**D**). CT, computed tomography

Model 1 (Table 5 and S8) showed good overall internal and external validation performance. The optimism-corrected c-index of the model was 0.68 (95% CI 0.62; 0.71). Discrimination was similar in both external validation cohorts: a c-index of 0.70 (95% CI 0.63; 0.77) for the cohort with non-injected scans and 0.66 (95% CI 0.59; 0.72) for the cohort with injected scans (Table 2, Fig. 2). Figure 2 C and D display the calibration graph of prediction models.

**Table 2:** Model performance across internal and external validation cohorts. Discriminative performance was measured using area under receiver operating characteristics curves and intercept

	Development cohort			External validation				
	Development cohort	Internal validation		CT without injection		CT with injection		
	Estimation	Estimation	95% CI	Estimation	95% CI		Estimation	95% CI
*C-index*								
Qualitative model/model 1	0.715	0.683	[0.621; 0.706]	0.702	[0.634; 0.769]		0.657	[0.589; 0.724]
Quantitative model/model 2	0.751	0.719	[0.666; 0.743]	0.730	[0.666; 0.795]		0.723	[0.660; 0.785]
Radiomics model/model 3	0.776	0.739	[0.694; 0.763]	0.767	[0.706; 0.827]		0.722	[0.659; 0.786]
Nagelkerke’s* R*^*2*^								
Qualitative model/model 1	0.172	0.089	NA	0.163	NA		0.114	NA
Quantitative model/model 2	0.212	0.117	NA	0.211	NA		0.192	NA
Radiomics model/model 3	0.265	0.196	NA	0.263	NA		0.146	NA

*CI* confidence interval, *CT* computed tomography, *NA* not applicable

### Performance of the quantitative model (model 2)

Using CT quantification (Table 5 and S8) improved the discrimination of the clinical prediction model up to a c-index of 0.72 (95% CI, 0.67; 0.74). The improvement from model 1 to model 2 was 0.04 (95% CI, 0.01; 0.07). Discrimination was similar in both external validation cohorts: a c-index of 0.73(95% CI 0.67; 0.80) with non-injected scans and 0.72(95% CI 0.66; 0.79) with injected scans (Table 2, Fig. 2).

A risk threshold of 0.49 was selected to achieve a specificity of at least 90% [6, 7] in the development cohort. Using this threshold for identification of high-risk population in the validation cohorts, sensitivity and negative predictive value were improved (0.23, 95% CI: 0.14; 0.32 and 0.07, 95% CI: 0.02; 0.11) at the expense of specificity (− 0.09, 95% CI: − 0.16; − 0.03) in comparison with model 1 in the cohort with injected scans (Table S4). The adjusted association of predictors with the outcome is detailed in Table 5.

### Performance of the model 3

In addition to the percentage of consolidation, six radiomics features were selected (namely volume of lesion and shape sphericity of lungs ROI from the shape groups, first order_Energy for lesion and lungs ROIs from the first order groups, and gldm_Dependence Entropy from lesion ROI, ngtdm_Busyness from lungs ROI from the second order groups, using pyradiomics canonical names). Using these 6 selected radiomics features instead of CT scan quantification of ground-glass and low attenuation areas (Table 5 and S8, Figure S2) improved slightly discrimination of the prediction model (optimism-corrected c-index 0.74, 95% CI: 0.69; 0.76). The improvement from model 1 to model 3 was 0.06 (95% CI, 0.03; 0.10). However, the improvement from model 2 to model 3 was not significant at 0.02 (95% CI, 0.00; 0.05). Discrimination was consistent in both external validation cohorts, although slightly lower among patients with injected scans: c-index of 0.77 (95% CI 0.71; 0.83) with non-injected scans and 0.72 (95% CI 0.66; 0.79) with injected scans (Table 2, Fig. 2).

Similarly, a risk threshold of 0.52 was selected to achieve a specificity of at least 90% in the development cohort. In the cohort with non-injected scans, sensitivities and negative predictive values were improved in comparison with models 1 and 2 (0.17, 95% CI: 0.07; 0.27, 0.06, 95% CI: 0.01; 0.11, 0.17, 95% CI: 0.08; 0.27 and 0.04, 95% CI: 0.01; 0.09 respectively), at the expense of specificity (− 0.08, 95% CI: − 0.15; − 0.02) in comparison with model 2. In the cohort with injected scans, sensitivity was increased (0.10, 95% CI: 0.00; 0.23) in comparison with model 1 whereas sensitivity was decreased (− 0.12, 95% CI: − 0.20; − 0.05) and specificity was increased (0.08, 95% CI: 0.03; 0.14) in comparison with model 2 (Table S4).

### Performance of the machine learning model

Using the development cohort, we selected the procedure (imputation, feature selection, oversampling classification) that gave the best mean c-index (Table 3, Table S6). We only show results for feature selection by taking the 50 best features after univariate analysis, taking a fixed percentile of features, or keeping the first components of a PCA yield inferior or similar c-index. We retrained the best-selected procedure on the whole development cohort. A threshold was selected for predictions to achieve a specificity of at least 90% in the development cohort. We obtained a c-index similar to the ones of the development cohort, which may hint at a good generalization ability of this model (Table 4). To analyze multi-centric variability, we have also evaluated the results on each center of the development cohort with no significant difference (Table S7). The ablation study (Table S5) shows the interest of considering imaging features from the parenchyma in addition to those from the lesion. Yet the results of this model are a bit worse than model 3 (Table 5).

**Table 3:** Results obtained with various machine learning procedures (imputation, feature selection, oversampling classification) on the development cohort. We selected the model with the best mean c-index over 30 repeated tenfold cross-validations

Procedure/metrics	Balanced accuracy	F1	Brier score	Precision	Recall	C-index
Vote (SVM, LR)	0.66 [0.6545, 0.6668]	0.56 [0.5523, 0.5663]	0.21 [0.2097, 0.2140]	0.48 [0.4738, 0.4869]	0.68 [0.6659, 0.6877]	0.73 [0.7208, 0.7328]
Hgboost	0.61 [0.6036, 0.6168]	0.47 [0.4623, 0.4814]	0.25 [0.2474, 0.2552]	0.47 [0.4638, 0.4818]	0.48 [0.4661, 0.4900]	0.70 [0.6889, 0.7013]
SVM	0.65 [0.6484, 0.6602]	0.56 [0.5502, 0.5630]	0.21 [0.2108, 0.2153]	0.46 [0.4570, 0.4685]	0.71 [0.6949, 0.7168]	0.72 [0.7180, 0.7302]
Random forests	0.65 [0.6407, 0.6530]	0.54 [0.5369, 0.5511]	0.21 [0.2099, 0.2140]	0.47 [0.4591, 0.4717]	0.66 [0.6507, 0.6732]	0.72 [0.7091, 0.7218]
LR	0.66 [0.6554, 0.6675]	0.56 [0.5536, 0.5672]	0.21 [0.2088, 0.2130]	0.48 [0.4745, 0.4874]	0.68 [0.6679, 0.6891]	0.73 [0.7233, 0.7358]

*CI* confidence interval, *LR* logistic regression, *SVM* support vector machine, *Hgboost* histogram-based gradient boosting classification tree, *Vote* soft vote of the SVM and LR classifiers

**Table 4:** Performance of the machine learning model on the two validation cohorts. Cutoff value for predictions was selected to ensure a specificity above .9 on the development cohort

Cohort/metrics	Balanced accuracy	F1	Brier score	Precision	Recall	C-index
Injected	0.67 [0.6649, 0.6686]	0.62 [0.6189, 0.6239]	0.24 [0.2422, 0.2439]	0.62 [0.6180, 0.6240]	0.62 [0.6211, 0.6270]	0.72 [0.7160, 0.7201]
Non-injected	0.64 [0.6380, 0.6419]	0.54 [0.5418, 0.5476]	0.22 (0.2171, 0.2187]	0.65 [0.6484, 0.6557]	0.47 [0.4668, 0.4732]	0.74 [0.7411, 0.7453]

**Table 5:** Association of each predictor with the outcome in models 1, 2, and 3

Models	Predictor	OR	95% CI	*p*-value
*Model 1*				
	Age (+ 1 year)	1.03	[1.01; 1.04]	< 0.0001
	Gender (female vs male)	0.74	[0.53; 1.05]	0.088
	Lesion extent (moderate vs mild) on CT scan	2.26	[1.35; 3.79]	< 0.0001
	Lesion extent (extended vs mild)	3.53	[2.02; 6.16]	
	Lesion extent (severe vs mild)	6.96	[3.37; 14.4]	
	Active smoking	0.59	[0.30; 1.16]	0.128
	Time elapsed since the onset of symptoms (+ 1 day)	0.97	[0.94; 1.00]	0.026
	Pre-existing cardiovascular diseases	1.08	[0.74; 1.57]	0.690
	Obesity	1.63	[1.07; 2.48]	0.024
	Pre-existing respiratory disease (COPD or ILD)	1.26	[0.80; 1.97]	0.317
	Diabetes	1.27	[0.85; 1.89]	0.246
	Immunosuppression	1.32	[0.76; 2.28]	0.330
	CRP (+ 1 mg/L)	0.38	[0.03; 4.11]	0.423
	Lymphocytes (+ 1 G/L)	1.02	[0.90; 1.15]	0.743
*Model 2*				
	Age (+ 1 year)	1.02	[1.01; 1.04]	0.0005
	Gender (female vs male)	0.79	[0.55; 1.13]	0.1929
	Ground glass extent (+ 5%)	1.19	[1.10; 1.28]	< .0001
	Consolidation (+ 5%)	1.40	[1.02; 1.91]	0.038
	Low attenuation areas below − 950 HU (LAA-950) (+ 5%)	1.21	[0.81; 1.82]	0.352
	Active smoking	0.63	[0.32; 1.23]	0.176
	Time elapsed since the onset of symptoms (+ 1 day)	0.97	[0.94; 0.99]	0.018
	Pre-existing cardiovascular diseases	1.06	[0.72; 1.55]	0.773
	Obesity	1.48	[0.96; 2.28]	0.078
	Pre-existing respiratory disease (COPD or ILD)	1.17	[0.73; 1.87]	0.511
	Diabetes	1.23	[0.82; 1.86]	0.324
	Immunosuppression	1.31	[0.74; 2.29]	0.351
	CRP (+ 1 mg/L)	0.45	[0.06; 3.68]	0.458
	Lymphocytes (+ 1 G/L)	1.03	[0.91; 1.16]	0.637
*Model 3*				
	Age (+ 1 year)	1.02	[1.01; 1.04]	0.002
	Gender (female vs male)	0.78	[0.54; 1.14]	0.203
	Volume of COVID lesions	0.15	[0.01; 2.94]	0.213
	Consolidation (+ 0.01 unit)	1.01	[0.94; 1.09]	0.822
	1st order energy (+ 1 trillion units)	2.02	[0.52; 7.82]	0.306
	Entropy (+ 0.1 unit)	1.21	[1.10; 1.32]	< 0.001
	Sphericity (+ 0.1unit)	0.53	[0.32; 0.85]	0.009
	1st order energy in lungs (+ 10 billion units)	0.96	[0.93; 0.98]	0.001
	Agitation (+ 1000 units)	1.14	[0.48; 2.69]	0.768
	Active smoking	0.72	[0.35; 1.48]	0.370
	Time elapsed since the onset of symptoms (+ 1 day)	0.96	[0.93; 0.99]	0.011
	Pre-existing cardiovascular diseases	1.19	[0.80; 1.76]	0.395
	Obesity	1.60	[1.02; 2.51]	0.040
	Pre-existing respiratory disease (COPD or ILD)	1.30	[0.81; 2.07]	0.273
	Diabetes	1.19	[0.78; 1.81]	0.427
	Immunosuppression	1.29	[0.72; 2.30]	0.395
	CRP (+ 1 mg/L)	1.01	[0.99; 1.03]	0.469
	Lymphocytes (+ 1 G/L)	1.06	[0.94; 1.20]	0.327

*CI* confidence interval, *COPD* chronic obstructive pulmonary disease, *CRP* C-reactive protein, *ILD* interstitial lung disease, *HU* Hounsfield Unit, *OR* odds ratio

## Discussion

While the COVID-19 pandemic is not over yet, identifying at-risk of worsening patients from mild COVID-19, by developing easy-to-use prediction models, remains a major issue, especially for potential new patient management strategies. Here, qualitative CT scan analyses combined with simple clinical and biological parameters could predict the worsening of COVID-19 pneumonia from mild forms with a c-index of 0.70. Using CT scan quantification improves the discrimination of the prediction model up to 0.73 and radiomics data up to 0.77. Discrimination was similar in both external validation cohorts with non-injected and injected CT scans. We also defined thresholds with high specificity in order to avoid false positive findings in order to optimize healthcare resources and/or to screen patients who would undergo new therapeutic options.

One may suggest that the prediction of clinical deterioration could be disappointing. However, similar data in more severe COVID-19 population reached the same performance of predicted clinical deterioration towards critical forms at day 14, varying from c-index 0.70 (95% CI 0.68; 0.72) to 0.78 (95% CI 0.74; 0.82) [11, 32]. Even when adding blood and physiological parameters, prognosis performance modestly improved discrimination (c-index = 0.735; 95% CI 0.715; 0.75) [21]. More recently, Davies et al developed a model to predict the need for intensive oxygen supplementation during hospitalization, including seven clinical and biological variables [22]. However, contrary to our study, validation on an external cohort was missing, probably inducing overestimated results. Kamran et al developed another model based on nine clinical characteristics which achieved a c-index of 0.80 (95% CI 0.77; 0.84). Performance was consistent when validated in external centers [23]. However, similar to most published studies, these patients, all needing hospitalization, are more severe than those selected in the present study, probably explaining these discrepancies [14, 33]. Besides, we have also decided to select easily available clinical and biological data to improve the feasibility of our models in the future. Prediction performance remained consistent despite temporal changes in management and treatment during the different COVID-19 waves. Application within the validation cohorts shows that this tool could guide clinician decisions, including treatment escalation.

Most of the already reported prediction scores were built on hospitalized cohort with more severe forms than our cohort, in addition to the use of a large number of parameters that are not systematically recorded in routine [8, 21–23]. We paid a particular attention to only include mild forms of COVID-19 and to use in our prediction model simple clinical and biological parameters along with chest CT data. Indeed, CT, apart from precluding thromboembolism complication, might have a predictive value on the progression to moderate/severe forms of COVID, helping for the development of new strategies.

One of the strengths of the present study was to compare the performance of two validation cohorts: first among patients with non-injected CT scans and second with injected CT scans. Interestingly, discrimination performance was similar in both external validation cohorts. We only noticed a decrease in discrimination performance in radiomics model. These results are important as an injection is now recommended regarding thromboembolism risk [8], which can occur even in mild COVID-19 [9] and will help to extrapolate our prediction model to larger real-life cohorts.

The frequent use of corticosteroids, based on its interest in lowering 28-day mortality, among patients with severe forms of COVID-19 [34, 35] but also in milder forms [36] must be considered. Indeed, patients from the validation cohort were more often treated with corticosteroids, as included later in the pandemic (Figure S1). The prediction performance of our models remains similar even though the therapeutic management of COVID-19 has improved, which supports the robustness of this model.

Contrary to previously published data [15], AI-enhanced imaging and clinical and biological information did not significantly improve the capacity to identify patients with pejorative outcomes. Direct comparison is difficult as we used a different dataset. External validation using an independent dataset is critical before implementation in a real-world environment and has been performed in the present study. Besides, opaque machine-learning algorithm black-box models have been avoided as much as possible by controlling valid clinical endpoints.

### Limitations

First, other clinical and biological characteristics not always available in standard practice [21–23] have been described as predictors of adverse outcomes, although in severe COVID-19 population. We thus decided not to include all these parameters in our predictive model which focuses on ambulatory patients. Second, the biological confirmation of COVID-19 was not systematically available, with 35% not having an initial positive RT-PCR. This might have negatively affected our evaluation, since several patients with negative RT-PCR but positive CT findings were considered having COVID-19. However, this limitation reflects real-life events where RT-PCR is not performed systematically in outdoor patients. Furthermore, previous data have shown that patients with a negative first RT-PCR test do not differ considering mortality or hospital stay length [37]. Besides, we selected patients with highly suspicious or compatible lesions on scans. Although the models showed consistent performance across five various centers, the ongoing performance of our models will need to be assessed in the context of increasing deployment of immunomodulatory agents [7, 38] and COVID-19 vaccines, as well as emerging SARS-CoV-2 variants.

## Conclusion

Models to predict clinical deterioration from mild to moderate forms were developed in response to the COVID-19 pandemic at five different hospitals, and were applied externally and performed well across the different medical centers, showing its potential as a tool for use in optimizing healthcare resources and selecting at-risk patients for new therapeutic strategies. Qualitative CT scan analyses combined with simple clinical and biological parameters could predict the worsening of COVID-19 pneumonia. The use of CT scan quantification or radiomics increased the performance of this prediction model.

### Supplementary information

Below is the link to the electronic supplementary material.

Supplementary file1 (PDF 614 KB)

## Abbreviations

BMI: Body mass index
CI: Confidence interval
COPD: Chronic obstructive pulmonary disease
COVID-19: Coronavirus disease 2019
CRP: C-reactive protein
CT: Computed tomography
DLP: Dose length product
HU: Hounsfield Unit
ILD: Interstitial lung disease
kV: Kilovoltage
LR: Logistic regression
mAs: Milliampere second
NA: Not applicable
OR: Odds ratio
SD: Standard deviation
SVM: Support vector machine

## Declarations

### Guarantor

The scientific guarantor of this publication is Pr Francois Laurent.

### Statistics and biometry

Julien Asselineau, Eric Frison, and Rodolphe Thiebaut kindly provided statistical advice for this manuscript.

### Study subjects or cohorts overlap

No study subjects or cohorts have been previously reported.

### Methodology

- retrospective
- diagnostic
- multicenter study
