# Supplementary material for: Development and external validation of a prediction model for the transition from mild to moderate or severe form of COVID-19
Source: Eur Radiol. 2023 Jul 5;33(12):9262–74. doi: 10.1007/s00330-023-09759-x (PMC10667132; doi:10.1007/s00330-023-09759-x)
Supplement: Supplementary file 1 — Supplementary file1 (PDF 614 KB) [file 330_2023_9759_MOESM1_ESM.pdf]

ELECTRONIC SUPPLEMENTARY MATERIAL

Development and external validation of a prediction model for the worsening of mild forms of COVID-19

Methods

Ethics considerations

The study was conducted by international guidance, including the Good Clinical Practices and the Declaration of Helsinki. According to national laws, the study was approved by a national Ethics Committee (Ethics and Scientific Committee for Research, Studies and Evaluations in the Health Field, CESREES) on 06/18/2020 (NCT04481620). All surviving participants at the data collection date have been provided comprehensive information notice regarding the purpose of the study, possible risks and expected benefits of the study, and participant's rights (voluntary participation and freedom to decline participation in the trial). The data recorded during the study have been collected, stored, and analyzed per the data protection laws in force.

All collected data were pseudonymized so that data will never indirectly mention the names of the persons concerned or their addresses. Only the information strictly necessary for the processing and the purpose of the study was collected.

All investigators conducted the study in adherence to:

- The principle of respect for human dignity and the principles of non-exploitation, non-discrimination and non-instrumentalization,
- The principle of individual autonomy (entailing the giving of free and informed consent, and respect for privacy and confidentiality of personal data),
- The principle of justice and the principle of beneficence and non-maleficence, namely with regard to the improvement and protection of health,
- The principle of proportionality (including that research methods are necessary to the aims pursued and that no alternative more acceptable methods are available).

CT scans

The two cohorts' different characteristics of the CT scans are detailed in the supplemental Table S1. All images were fully pseudonymized, including the date of the CT examination (supplemental data methods). For half of the external validation cohort (n=246), a CT pulmonary angiography (CTPA) was also performed.

For the injected CT scans, patients received 2 mL/kg (maximum 140 mL) of contrast material (Xenetix 350, Guerbet, Aulnay, France or Ioméron®, Ioméprol, Bracco Imaging France, Ultravist®, Iopromide, Bayer Healthcare, SAS) followed by a saline chaser of 30 mL, delivered at 4 mL/s. The acquisition was initiated using an automatic bolus tracking technique with a region of interest positioned at the level of the main pulmonary artery and a threshold CT value preset at 110 HU.

from ground glass opacities. In addition to COVID-19-related quantitative parameters, relative low attenuation areas below -950 HU (LAA-950HU) were quantified from the segmented lung CT images using in-house software written in Matlab (Natick, Massachusetts: The MathWorks Inc.) as a surrogate for emphysema extension. Emphysema was considered present when the percentage of LAA-950HU was higher than 6% [1].

The standardized report proposed by the French Society of Radiology assesses the severity of COVID-19 on chest CT scans was largely used by French radiologists across the different participating centers of this study. In case of the standardized reporting was lacking, the CT images were reviewed by a senior radiologist with over 30 years of experience in chest imaging (FL).

The percentages of opacity and high opacity (relative to the lung volume) were calculated. A threshold of −100 HU was applied inside the detected opacities to separate high opacities (consolidations).

Development and validation of the prediction model

The selection procedure was the following: for COVID and lungs ROIs, we picked the best features from the univariate analysis of the development cohort in each of the classical radiomics classes (shape-based features, first-order intensity features, and second-order intensity features). This yields 6 features, namely volume of lesion and shape_sphericity of lungs ROI from the shape groups, first order_Energy for lesion and lungs ROIs from the first order groups, and gldm_Dependence Entropy from lesion ROI, ngtdm_Busyness from lungs

ROI from the second order groups, using pyradiomics canonical names. In addition, we included the percentage of consolidation as the seventh feature.

The functional form of association of quantitative predictors with the outcome was evaluated by the 2-degrees fractional polynomials method needed in a resampling procedure (n=1,000) to ensure robust choice of the forms [2].

Criteria were aggregated by using the median on each imputed dataset (2). Study center effect on predictive performances was not directly tackled but within-study centers performances were studied as sensitivity analyses [4].

To correct the performance criteria for optimism (internal validation), we applied the 0.632+ bootstrap method [5] . To combine bootstrapping and multiple imputation, the “Val-MI” strategy was adopted with 1,000 bootstrap resamples and only one imputation [6]. Confidence intervals were calculated with an approach derived from Jiang *et al.* [6, 7]. Internal validation process was performed on c-index, difference of c-index between clinical prediction models and the scaled Brier score.

Development and validation

According to Rubin's rule, the missing data on outcome and predictors (Table S2) were handled with a multiple imputation method using a fully conditional specification method [2, 8], twenty imputed datasets and pooling results across imputations. The study center was introduced into the model as a random effect [4]. To limit overfitting and optimism in performance of prediction models [9, 10], no selection procedure was applied. Discriminative ability was assessed using Harrell's c-index and Nagelkerke’s R^2. Internal validation methodology is detailed in supplemental methods.

Clinical prediction models were applied to participants to estimate their outcome probability. C-indexes and difference of C-indexes between models were calculated with their two-sided 95% confidence interval .

Development and validation of machine learning model

Preprocessing, harmonization and normalization of features: radiomics features were scaled using the RobustScaler from scikit-learn, which removes the median and scales the data according to the quantile range. Categorical features were one-hot encoded. Missing categorical features were imputed using the most frequent value, missing numerical features with a KNN imputer with K=5. Multivariate imputation (Iterative Imputer from [12]) was also investigated without any gain in score.

Data oversampling to tackle dataset imbalance: We used the SMOTE algorithm [13]. Training a machine learning algorithm and evaluating its performance on the development cohort. Several methods were investigated (logistic regression, random forests, support vector machine, gradient boosting).

Supplemental tables

Supplemental Table S1: Characteristics of CT scans

Cohorts	Machine Brand	n	Kernels	kV	mAs	DLP	Slice thickness	Pixel size
						(mGy*cm)	(mm)	(mm^2)
**Development**	General Electric, GE® (Optima CT660, Revolution Frontier, Revolution HD, Revolution CT, Discovery CT750HD) ®	745 (295, 233, 172, 43, 2)	Soft (Standard, br40d, FC08, FC18, FC30)	(80-150)	(50-2399)	17-764	0.625-2.5	0.48-0.97
	Siemens Somatom (Force) ®	27						
	Philips (Ingenuity Core) ®	24						
	Toshiba (Aquilon Prime)®	16						
	Canon (Aquilon One)®	15						
**Validation**	Toshiba (Aquilon Prime)®	461	Soft (Standard, FC02, FC07, I30s)	(100-135)	(50-600)	8.4-568	1-2	0.54-0.97
	General Electric, GE® (Optima CT540) ®	10						
	Siemens Somatom (Scope) ®	1						
	Canon (Aquilon One)®	2						

Legends: kV; kilovoltage, mAs; milliampere second, DLP; Dose length product

Supplemental Table S2: Proportion of missing data for predictors and outcome in the study cohorts.

	**Development cohort (n=827)**	**Validation cohort (n=474)**	
		CT without injection (n=228)	CT with injection (n=246)
Predictors			
Age	0 (0%)	0 (0%)	0 (0%)
Gender	0 (0%)	0 (0%)	0 (0%)
Time to symptoms onset	1 (0%)	0 (0%)	0 (0%)
Active smokers	295 (35.7%)	81 (35.5%)	68 (27.6%)
Pre-existing cardiovascular diseases	0 (0%)	0 (0%)	0 (0%)
Obesity	5 (0.6%)	28 (12.3%)	57 (23.2%)
Pre-existing respiratory diseases	0 (0%)	0 (0%)	0 (0%)
Diabetes	0 (0%)	0 (0%)	0 (0%)
Immunosuppression	0 (0%)	0 (0%)	0 (0%)
Lymphocyte level	10 (1.2%)	7 (3.1%)	6 (2.4%)
CRP	10 (1.2%)	8 (3.5%)	3 (1.2%)
Disease extent on CT-scan	0 (0%)	0 (0%)	0 (0%)
Quantitative outcomes			
Outcome			
Significant clinical deterioration in the 30 days	81 (9.8%)	6 (2.6%)	2 (0.8%)

CRP: C-reactive protein, CT: computed tomography

Supplemental Table S3: Patient characteristics in the development cohort

	Bordeaux University hospital (n=537)	Private Hospital in Bordeaux (n=39)	Grenoble University hospital (n=173)	Montpellier University hospital (n=178)
Clinical parameters				
Age (years), mean (SD)	65.7 (18.4)	63.9 (14.2)	65.6 (16.2)	64.7 (17.6)
Male gender, N (%)	315 (58.7)	28 (71.8)	107 (61.8)	45 (57.7)
BMI (kg/m²)	26.9 (6.3)	28.1 (5.8)	28.6 (6.4)	28.1 (6.0)
Time to symptoms onset (days), mean (SD)	6.5 (7.9)	6.3 (5.7)	9.2 (9.5)	9.8 (7.6)
Active Smokers, N (%)	63 (11.7)	2 (5.1)	9 (5.2)	3 (3.8)
Hypertension, N (%)	251 (46.7)	14 (35.9)	77 (44.5)	31 (39.7)
Coronary artery disease, N (%)	107 (19.9)	6 (15.4)	33 (19.1)	19 (24.4)
Obesity, N (%)	103 (19.2)	12 (35.3)	41 (23.7)	22 (28.2)
Respiratory diseases				
Asthma, N (%)	489 (91.1)	37 (94.9)	153 (88.4)	71 (91.0)
COPD, N (%)	43 (8.0)	6 (15.4)	13 (7.5)	8 (10.3)
Interstitial lung disease, N (%)	12 (2.2)	0 (0.0)	4 (2.3)	1 (1.3)
Diabetes, N (%)	111 (20.7)	4 (10.3)	39 (22.5)	16 (20.5)
Immunosuppression, N (%)	58 (10.8)	1 (2.6)	13 (7.5)	11 (14.1)
Biological Parameters				
Lymphocyte level (g/L)	1.15 (1.59)	1.28 (0.75)	1.23 (0.76)	1.06 (0.55)
CRP (mg/L)	86.2 (72.7)	67.9 (64.1)	82.7 (73.2)	107.9 (144.8)
RT-PCR positive for COVID-19, N (%)	250 (57.1)	24 (66.7)	124 (77.5)	63 (81.8)
Radiological Parameters				
Disease extent on CT-scan				
Mild <10%, N (%)	121 (22.5)	8 (20.5)	42 (24.3)	11 (14.1)
Moderate 10-25%, N (%)	268 (49.9)	13 (33.3)	78 (45.1)	30 (38.5)
Extended 25-50%, N (%)	114 (21.2)	14 (35.9)	41 (23.7)	31 (39.7)
Severe 50-75%, N (%)	31 (5.8)	4 (10.3)	12 (6.9)	5 (6.4)
Critical >75 %, N (%)	3 (0.6)	0 (0.0)	0 (0.0)	1 (1.3)
Outcomes				
Primary Outcome in the 30 days	144 (28.1)	12 (32.4)	56 (38.9)	29 (54.7)
Outcome				
Moderate form	124 (24.2)	11 (29.7)	50 (34.7)	27 (50.9)
Severe form	56 (10.9)	2 (5.4)	30 (20.8)	17 (32.1)
Critical form	14 (2.7)	2 (5.4)	20 (13.9)	10 (18.9)
Death	46 (9.1)	2 (5.4)	12 (9.2)	7 (14.3)

BMI: body mass index, CRP: C-reactive protein, COPD: Chronic Obstructive Pulmonary Disease, CT: computed tomography, RT-PCR: Real-Time Polymerase Chain Reaction, SD: standard deviation

Supplemental Table S4: Predictive performances in both external validation samples by using the thresholds defined to obtain a minimal specificity of 0.90

		External validation : CT without injection		External validation : CT with injection	
		Estimate	95%CI	Estimate	95%CI
Sensitivity	Qualitative model / Model 1	0.35	0.26;0.46	0.30	0.22;0.40
	Quantitative model / Model 2	0.35	0.25;0.45	0.53	0.43;0.63
	Radiomics model / Model 3	0.52	0.42;0.62	0.41	0.31;0.51
Difference of sensitivities	Model 2 – Model 1	-0.01	-0.09;0.08	0.23	0.14;0.32
	Model 3 – Model 1	0.17	0.07;0.27	0.10	0.00;0.23
	Model 3 – Model 2	0.17	0.08;0.27	-0.12	-0.20;-0.05
Specificities	Qualitative model / Model 1	0.82	0.75;0.88	0.84	0.76;0.89
	Quantitative model / Model 2	0.88	0.81;0.93	0.74	0.66;0.81
	Radiomics model / Model 3	0.79	0.71;0.86	0.82	0.75;0.88
Difference of specificities	Model 2 – Model 1	0.05	-0.02;0.12	-0.09	-0.16;-0.03
	Model 3 – Model 1	-0.03	-0.11;0.05	-0.01	-0.09;0.06
	Model 3 – Model 2	-0.08	-0.15;-0.02	0.08	0.03;0.14
Positive predictive value	Qualitative model / Model 1	0.60	0.47;0.73	0.57	0.43;0.71
	Quantitative model / Model 2	0.68	0.53;0.81	0.61	0.50;0.71
	Radiomics model / Model 3	0.65	0.54;0.76	0.63	0.51;0.75
Difference of PPV	Model 2 – Model 1	0.08	-0.04;0.20	0.03	-0.07;0.14
	Model 3 – Model 1	0.05	-0.06;0.16	0.06	-0.08;0.19
	Model 3 – Model 2	-0.03	-0.13;0.07	0.03	-0.04;0.10
Negative predictive value	Qualitative model / Model 1	0.63	0.56;0.70	0.61	0.54;0.68
	Quantitative model / Model 2	0.64	0.57;0.72	0.68	0.60;0.75
	Radiomics model / Model 3	0.69	0.61;0.76	0.65	0.57;0.73
Difference of NPV	Model 2 – Model 1	0.01	-0.02;0.05	0.07	0.02;0.11
	Model 3 – Model 1	0.06	0.01;0.11	0.04	-0.01;0.08
	Model 3 – Model 2	0.04	0.01;0.09	-0.03	-0.07;0.01

95%CI : two-sided 95% confidence interval

Supplemental Table S5: Ablation study: performances for different subsets of features

Features / Metrics	Cohort	Balanced Accuracy	F1	Brier Score	Precision	Recall	c-index
Lesion radiomics	Injected	0.66 [0.6561, 0.6598]	0.63 [0.6254, 0.6301]	0.26 [0.2571, 0.2590]	0.59 [0.5869, 0.5925]	0.67 [0.6706, 0.6762]	0.69 [0.6921, 0.6964]
Lesion radiomics	Non-Injected	0.62 [0.6176, 0.6215]	0.51 [0.5105, 0.5164]	0.22 [0.2227, 0.2242]	0.63 [0.6220, 0.6296]	0.44 [0.4344, 0.4406]	0.71 [0.7094, 0.7139]
Lesion + lung radiomics	Injected	0.69 [0.6853, 0.6890]	0.66 [0.6553, 0.6599]	0.25 [0.2499, 0.2518]	0.62 [0.6176, 0.6232]	0.70 [0.6990, 0.7045]	0.72 [0.7166, 0.7207]
Lesion + lung radiomics	Non-injected	0.65 [0.6490, 0.6530]	0.58 [0.5728, 0.5785]	0.23 [0.2245, 0.2261]	0.64 [0.6375, 0.6444]	0.52 [0.5216, 0.5278]	0.73 [0.7286, 0.7328]
Complete model	Injected	0.69 [0.6853, 0.6890]	0.66 [0.6553, 0.6599]	0.25 [0.2499, 0.2518]	0.62 [0.6176, 0.6232]	0.70 [0.6990, 0.7045]	0.72 [0.7166, 0.7207]
Complete model	Non-injected	0.65 [0.6490, 0.6530]	0.58 [0.5728, 0.5785]	0.23 [0.2245, 0.2261]	0.64 [0.6375, 0.6444]	0.52 [0.5216, 0.5278]	0.73 [0.7286, 0.7328]

Supplemental Table S6: Parameters of the machine-learning classification algorithm (implementations from Scikit-Learn https://scikit-learn.org)

**Algorithms**	**Parameters**
SVM	gamma=1e-6, C=2e3, class_weight='balanced'
Random Forest	n_estimators = 150, bootstrap=True, max_depth=4
LR	C=0.01
Hgboost	l2_regularization=1.0, learning_rate=0.01, max_depth=15
Vote (SVM, LR)	Soft vote with the SVM and LR classifiers described above.

LR: Logistic Regression, SVM: Support Vector Machine, Hgboost: Histogram-based gradient boosting classification tree, Vote: soft vote of the SVM and LR classifiers.

Supplemental Table S7: Performance on each center of the development cohort (best procedure trained on all groups of the development cohort but the one under investigation)

Center / Metrics	Balanced Accuracy	F1	Brier Score	Precision	Recall	c-index
**Bordeaux University hospital**	0.67 [0.6661, 0.6688]	0.53 [0.5303, 0.5340]	0.20 [0.2025, 0.2034]	0.43 [0.4238, 0.4278]	0.71 [0.7094, 0.7140]	0.76 [0.7575, 0.7602]
**Private Hospital in Bordeaux**	0.59 [0.5822, 0.5919]	0.39 [0.3808, 0.3980]	0.20 [0.2023, 0.2063]	0.49 [0.4812, 0.5039]	0.34 [0.3302, 0.3476]	0.66 [0.6491, 0.6625]
**Grenoble University hospital**	0.73 [0.7279, 0.7325]	0.68 [0.6795, 0.6854]	0.19 [0.1901, 0.1917]	0.63 [0.6256, 0.6328]	0.75 [0.7460, 0.7530	0.79 [0.7895, 0.7941]
**Montpellier University hospital**	0.76 [0.7611, 0.7684]	0.79 [0.7910, 0.7981]	0.22 [0.2174, 0.2202]	0.77 [0.7647, 0.7742]	0.83 [0.8229, 0.8314]	0.74 [0.7315, 0.7410]

Table S8: Details of the prediction models with all regression coefficients and model intercept.

	**Qualitative model Model 1**	**Quantitative model Model 2**	**Radiomics model Model 3**
Intercept	-3.214	-2.773	-14.14
Age (+ 1 year)	0.026	0.023	0.021
Female gender (yes)	-0.299	-0.237	-0.245
Active smoking (yes)	-0.522	-0.467	-0.327
Time to symptoms onset (+1 day)	-0.031	-0.033	-0.039
Pre-existing cardiovascular disease (yes)	0.076	0.056	0.171
Obesity (yes)	0.486	0.390	0.470
Pre-existing respiratory disease (yes)	0.229	0.157	0.260
Diabetes (yes)	0.236	0.207	0.171
Immunosuppression (yes)	0.274	0.268	0.252
$1/CRP^2$ (+1 unit)	-0.976	-0.794	/
CRP (+10 G/L)	/	/	0.009
Lymphocytes (+1 G/L)	0.020	0.029	0.060
Qualitative CT-scan data			
Lesion extent			
Mild	0	/	/
Moderate	0.817	/	/
Extended	1.261	/	/
Severe	1.940	/	/
Quantitative CT-scan data			
Ground glass extent (+5%)	/	0.170	/
Consolidation (+5%)	/	0.334	/
Low attenuation areas below -950 HU (LAA-950) (+5%)	/	0.192	/
Radiomics CT-scan data			
Volume of COVID lesions	/	/	-1.869
Consolidation (+0.01 unit)	/	/	0.009
1st order energy (+1 trillion units)	/	/	0.705
Entropy (+0.1 unit)	/	/	0.190
Sphericity (+0.1 unit)	/	/	-0.642
1st order energy in lungs (+10 billion units)	/	/	-0.044
Agitation (+1000units)	/	/	0.129

CRP: C-Reactive Protein, CT: computed tomography, HU: Hounsfield Unit

Supplemental Figures

Supplemental Figure 1

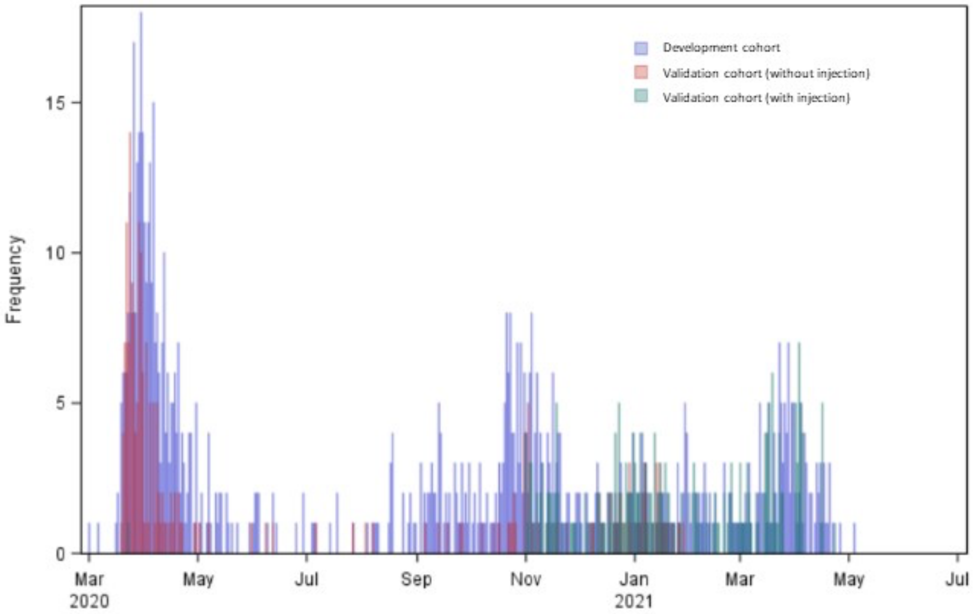

Figure S1: Inclusion distribution in the development and validation cohorts, between March 1, 2020 and April 31, 2021

Supplemental Figure 2

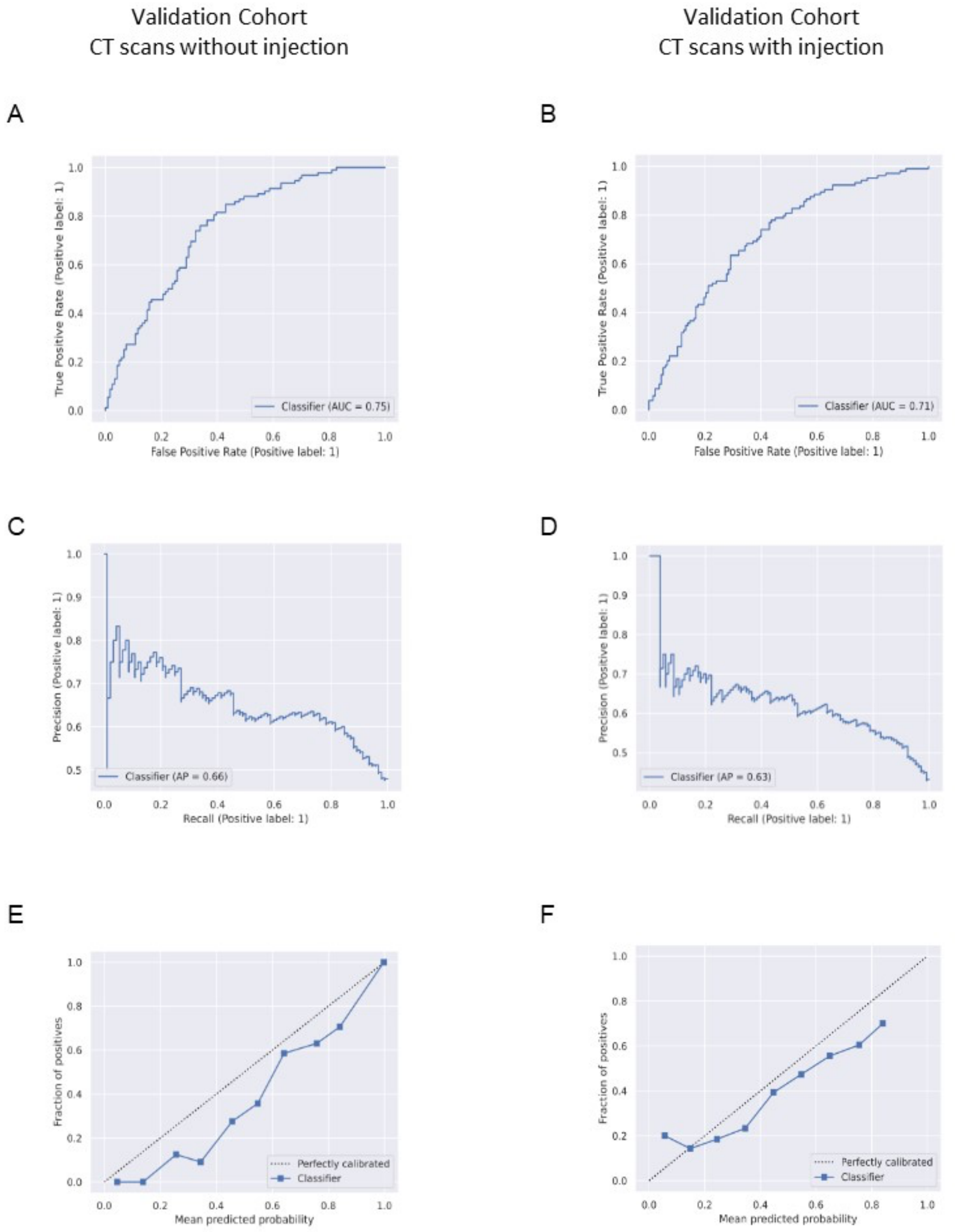

Figure S2: Performance of the radiomics model (i.e., Model 3), A.B c-index from the validation Cohort with CT scans respectively without (A) and with (B) injection, C, D recall from the validation Cohort with CT scans respectively without (C) and with (D) injection E, F calibration from the validation Cohort with CT scans respectively without (E) and with (F) injection

References

1. Lynch DA, Austin JHM, Hogg JC, et al (2015) CT-Definable Subtypes of Chronic Obstructive Pulmonary Disease: A Statement of the Fleischner Society. Radiology 277:192–205. https://doi.org/10.1148/radiol.2015141579

2. Royston P (2017) Model selection for univariable fractional polynomials. Stata J 17:619–629

3. Marshall A, Altman DG, Holder RL, Royston P (2009) Combining estimates of interest in prognostic modelling studies after multiple imputation: current practice and guidelines. BMC Med Res Methodol 9:57. https://doi.org/10.1186/1471-2288-9-57

4. Falconieri N, Van Calster B, Timmerman D, Wynants L (2020) Developing risk models for multicenter data using standard logistic regression produced suboptimal predictions: A simulation study. Biom J Biom Z 62:932–944. https://doi.org/10.1002/bimj.201900075

5. Efron, B., & Tibshirani, R. (1997). Improvements on cross-validation: the 632+ bootstrap method. Journal of the American Statistical Association, 92(438), 548-560

6. Wahl S, Boulesteix A-L, Zierer A, et al (2016) Assessment of predictive performance in incomplete data by combining internal validation and multiple imputation. BMC Med Res Methodol 16:144. https://doi.org/10.1186/s12874-016-0239-7

7. Jiang, B., Zhang, X., & Cai, T. Estimating the confidence interval for prediction errors of support vector machine classifiers. Journal of Machine Learning Research, 9(Mar), 521-540. 2008

8. van Buuren S (2007) Multiple imputation of discrete and continuous data by fully conditional specification. Stat Methods Med Res 16:219–242. https://doi.org/10.1177/0962280206074463

9. Harrell Jr, F. E. (2015). Regression modeling strategies: with applications to linear models, logistic and ordinal regression, and survival analysis. Springer;

10. Steyerberg, E. W. (2019). Clinical prediction models. Springer International Publishing

11. DeLong ER, DeLong DM, Clarke-Pearson DL (1988) Comparing the areas under two or more correlated receiver operating characteristic curves: a nonparametric approach. Biometrics 44:837–845

12. Abraham A, Pedregosa F, Eickenberg M, et al (2014) Machine learning for neuroimaging with scikit-learn. Front Neuroinformatics 8:14. https://doi.org/10.3389/fninf.2014.00014

13. Chawla, N. V., Bowyer, K. W., Hall, L. O., & Kegelmeyer, W. P. (2002). SMOTE: synthetic minority over-sampling technique. Journal of artificial intelligence research, 16, 321-357

Author contributions.

MZ, JA, OS, EF, RT, PB, FL, IB conceived the study.

MZ, JA, OS, EF, AM, JC, EK, RA, SR, SB, AB, GD, JC, AB, GF, BD, RT, PB, FL, IB contributed to the design and collection of data relating to the development cohort.
MZ, JA, OS, EF, MO, AM, FC, PB, FL, IB contributed to the design and collection of data relating to the validation cohort.
Data analysis was completed by JA, OS.
Interpretation and drafting of the manuscript were conducted by MZ, JA, OS, EF, PB, FL, IB.
All authors critically reviewed the manuscript and approved the final version.

Funding

This study was supported by the Interregional Hospital Program of Clinical Research ("Programme Hospitalier de Recherche Clinique Interregional" 2020, PHRCI 2020_20-016). The funder played no in the study or the preparation of the manuscript.

Availability of data and materials

The datasets used and analyzed during the current study are available from the corresponding author on reasonable request.
